# Supplementary material for: Mutations in PCYT2 disrupt etherlipid biosynthesis and cause a complex hereditary spastic paraplegia
Source: Brain. 2019 Oct 22;142(11):3382–97. doi: 10.1093/brain/awz291 (PMC6821184; doi:10.1093/brain/awz291)
Supplement: awz291_Supplementary_Data [file awz291_supplementary_data.zip › awz291-Suppl_data/Supplementary_Data4.zip!/Supplementary information 4 - plasma lipidomics.zip!/Plasma lipidomics/Control vs Patient/Statistical plots and data/Heatmap sorted on p-value and FC.pdf]

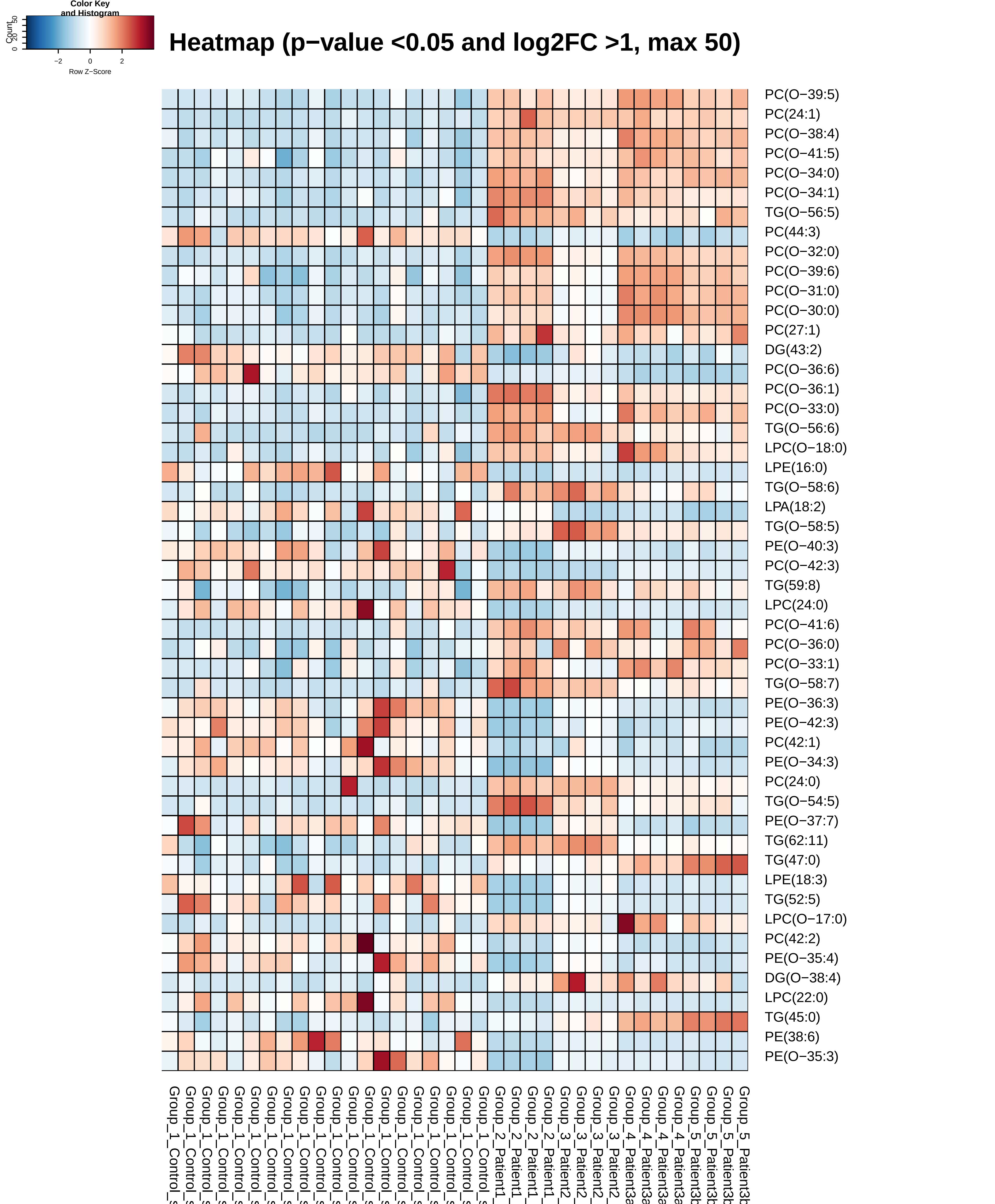

Color Key and Histogram
Count
Row Z−Score
Heatmap (p−value <0.05 and log2FC >1, max 50)
PC(O−39:5)
PC(24:1)
PC(O−38:4)
PC(O−41:5)
PC(O−34:0)
PC(O−34:1)
TG(O−56:5)
PC(44:3)
PC(O−32:0)
PC(O−39:6)
PC(O−31:0)
PC(O−30:0)
PC(27:1)
DG(43:2)
PC(O−36:6)
PC(O−36:1)
PC(O−33:0)
TG(O−56:6)
LPC(O−18:0)
LPE(16:0)
TG(O−58:6)
LPA(18:2)
TG(O−58:5)
PE(O−40:3)
PC(O−42:3)
TG(59:8)
LPC(24:0)
PC(O−41:6)
PC(O−36:0)
PC(O−33:1)
TG(O−58:7)
PE(O−36:3)
PE(O−42:3)
PC(42:1)
PE(O−34:3)
PC(24:0)
TG(O−54:5)
PE(O−37:7)
TG(62:11)
TG(47:0)
LPE(18:3)
TG(52:5)
LPC(O−17:0)
PC(42:2)
PE(O−35:4)
DG(O−38:4)
LPC(22:0)
TG(45:0)
PE(38:6)
PE(O−35:3)
Group_2_Patient1_
Group_3_Patient2_
Group_4_Patient3a
Group_5_Patient3b
